# A systematic review of lessons learned from PET molecular imaging research in atypical parkinsonism

**DOI:** 10.1007/s00259-016-3464-8

**Published:** 2016-07-28

**Authors:** Flavia Niccolini, Marios Politis

**Affiliations:** Neurodegeneration Imaging Group, Institute of Psychiatry, Psychology and Neuroscience (IoPPN), King’s College London, London, UK

**Keywords:** Atypical parkinsonism, Corticobasal syndrome, Multiple system atrophy, Progressive supranuclear palsy, Positron emission tomography

## Abstract

**Purpose:**

To systematically review the previous studies and current status of positron emission tomography (PET) molecular imaging research in atypical parkinsonism.

**Methods:**

MEDLINE, ISI Web of Science, Cochrane Library, and Scopus electronic databases were searched for articles published until 29th March 2016 and included brain PET studies in progressive supranuclear palsy (PSP), multiple system atrophy (MSA), and corticobasal syndrome (CBS). Only articles published in English and in peer-reviewed journals were included in this review. Case-reports, reviews, and non-human studies were excluded.

**Results:**

Seventy-seven PET studies investigating the dopaminergic system, glucose metabolism, microglial activation, hyperphosphorilated tau, opioid receptors, the cholinergic system, and GABA_A_ receptors in PSP, MSA, and CBS patients were included in this review. Disease-specific patterns of reduced glucose metabolism have shown higher accuracy than dopaminergic imaging techniques to distinguish between parkinsonian syndromes. Microglial activation has been found in all forms of atypical parkinsonism and reflects the known distribution of neuropathologic changes in these disorders. Opioid receptors are decreased in the striatum of PSP and MSA patients. Subcortical cholinergic dysfunction was more severe in MSA and PSP than Parkinson’s disease patients although no significant changes in cortical cholinergic receptors were seen in PSP with cognitive impairment. GABA_A_ receptors were decreased in metabolically affected cortical and subcortical regions in PSP patients.

**Conclusions:**

PET molecular imaging has provided valuable insight for understanding the mechanisms underlying atypical parkinsonism. Changes at a molecular level occur early in the course of these neurodegenerative diseases and PET imaging provides the means to aid differential diagnosis, monitor disease progression, identify of novel targets for pharmacotherapy, and monitor response to new treatments.

## Introduction

Atypical parkinsonism includes a heterogeneous group of movement disorders such as progressive supranuclear palsy (PSP), multiple system atrophy (MSA), and corticobasal syndrome (CBS). These disorders are characterised by the presence of parkinsonian symptomatology with poor response to levodopa and additional features beyond the classic Parkinson’s disease (PD) presentation.

MSA is an adult-onset, fatal neurodegenerative disease clinically characterized by a variable combination of progressive autonomic failure, parkinsonian features, and cerebellar and pyramidal features [1]. Its prevalence is about four to five cases per 100,000, and patients usually die after a median survival of 6 to 9 years [2]. MSA is pathologically characterised by glial, α-synuclein-positive, cytoplasmic inclusion (GCI) in affected brain regions including striatonigral and/or olivopontocerebellar systems [3].

PSP is a neurodegenerative disorder with prevalence of five to six cases per 100,000, with typical onset in the middle 60s and median survival of 7 years [4]. PSP is currently classified among the group of tau-positive frontotemporal lobar degenerations [5]. It is characterized clinically by the presence of symmetrical akinetic–rigid parkinsonian syndrome, supranuclear gaze palsy, early postural instability with falls backwards, subcortical dementia, dysarthria, and dysphagia [6, 7]. The pathological hallmark of PSP is accumulation of abnormal tau protein (4-repeat tau) in subcortical nuclei neurons forming the neurofibrillary tangles and glial cells as tufted astrocytes and oligodendrogial inclusions [8, 9].

CBS is a rare neurodegenerative disorder with prevalence ranging from 0.2 to four cases per 100,000 and an average time from symptoms onset to death of 7 years [10, 11]. The core clinical features of CBS include asymmetric rigidity and apraxia together with other features such as cortical sensory loss, alien limb behaviour, gaze palsy, atypical tremor, and dementia [12, 13]. CBS is characterized neuropathologically by deposits of 4-repeat tau in cortical and striatal neurons and glia, forming astrocytic plaques [14].

Early diagnosis of these three atypical parkinsonian syndromes is still challenging with 24 % of the patients being misdiagnosed [15]. Currently, there are no biomarkers or disease modifying therapy for these three disorders, while symptomatic treatment is very limited. Positron emission tomography (PET) molecular imaging is a powerful in vivo tool for investigating brain function such as metabolism, receptor, and enzyme distributions. PET is an analytical imaging method and has the potential to give both structural and kinetic information and in comparison with other imaging techniques, provides high sensitivity and temporal resolution [16]. In recent decades, PET imaging research has been employed extensively in movement disorders and has significantly contributed to progress in understanding atypical Parkinsonism (Table 1) [17–22]. Despite this progress only little of this understanding has been translated into the clinical setting [23, 24].

**Table 1:** Main PET molecular imaging changes in multiple system sclerosis, progressive supranuclear palsy, and corticobasal syndrome

	MSA	PSP	CBS
Brain Metabolism	Reduced brain metabolism in bilateral putamen, cerebellum, and brainstem	Reduced brain metabolism in bilateral medial frontal cortex, premotor areas, prefrontal areas, striatum (caudate in particular), thalamus, and brainstem	Asymmetric reduced brain metabolism contralateral to the most affected side involving parietal cortex, primary sensorimotor cortex, the medial and lateral premotor areas, striatum, and thalamus
Dopaminergic system	Reduced [^18^F]dopa uptake in caudate, putamen, ventral striatum, globus pallidus, and red nucleus. Reduced [^11^C]DTBZ uptake in caudate and putamen (also cerebellum in MSA-C)	Reduced [^18^F]dopa and [^18^F]FP-CIT uptake in caudate, putamen (caudate and anterior putamen affected at the same level as the posterior putamen)	Asymmetric decreased in [^18^F]Fdopa uptake in the caudate and the putamen contralateral to the most affected side
	Decreased striatal D2 receptor density	Decreased striatal D2 receptor density	
Microglial activation	Increased [^11^C]PK11195 binding was also found in the dorsolateral prefrontal cortex, caudate, putamen, pallidum, thalamus, substantia nigra, and pons	Increased [^11^C]PK11195 binding in caudate, putamen, pallidum, substantia nigra, midbrain, thalamus, cerebellum, and frontal lobe	Increased [^11^C]PK11195 binding in caudate, putamen, substantia nigra, and frontoparietal cortex
Tau deposition		Increased [^18^F]FDDNP binding in subthalamic area, midbrain region, and cerebellar white matter. Increased [^18^F]FDDNP binding in neocortical regions (frontal lobe, temporal lobe an posterior cingulate gyrus) only in PSP subjects with more severe disease	
Opioid system	Decreased opioid receptors in putamen, but not caudate of MSA-P	Decreased opioid receptors in both caudate and putamen	
	Decreased opioid receptors in both caudate and putamen in MSA-C		
GABAergic system		Decreased [^11^C]flumazenil binding in hypometabolic cortical regions	
Cholinergic markers	Decreased cortical and subcortical AChE activity in MSA-P	No significant changes in cortical mAChR levels in PSP patients with cognitive impairment.	
		Decreased subcortical AChE activity with greater involvement of the pontine cholinergic group.	

In this article, we aimed to review systematically the previous studies and current status of PET molecular imaging research in atypical parkinsonism. We reviewed disease-related patterns of PET molecular changes, their clinical correlates, and their value in aiding the differential diagnosis of atypical parkinsonism.

## Methods

### Search strategy

MEDLINE, ISI Web of Science, Cochrane Library, and Scopus databases electronic databases were searched for articles in English published until 29th March 2016. Gray literature (i.e., abstracts or conference proceedings), reviews, case-reports, and non-humans studies were not considered as a priority asset of our systematic review. Studies were identified, combining the following major Medical Subject Headings: “Atypical Parkinsonism” or “Corticobasal Degeneration” or “Multiple System Atrophy” or “Progressive Supranuclear Palsy” and “PET” combined with text and keywords (MEDLINE, for example): ([“Atypical Parkinsonism”, MeSH Terms, OR “Progressive Supranuclear Palsy” OR “Richardson-Steele-Olszewski syndrome” OR “Multiple System Atrophy”, MeSH Terms, OR “Shy-drager syndrome” OR “olivopontocerebellar atrophy” OR “striatonigral degeneration” OR “Corticobasal Ganglionic Degeneration” OR “Corticobasal Degeneration”] AND ]“Positron-Emission Tomography”, MeSH Terms, OR “positron emission tomography” OR “PET”]). Additional eligible studies were identified screening the reference lists of studies included in our analysis.

### Inclusion criteria

All selected titles and abstracts were independently reviewed by two authors (FN, MP). Studies were excluded if the title and/or abstract were not appropriate for the aim of the review. Full texts were subsequently obtained for eligible studies or when the relevance of an article could not be certainty excluded. Selected studies were eligible if they met the following criteria: 1) cross-sectional, case control, or longitudinal brain PET studies including PSP, MSA, and CBS patients; 2) published in peer-reviewed journals. Reviews, case-reports and non-human studies were excluded. PET measures included the following radioligands: [^11^C]raclopride (dopamine D2 receptors), [^11^C]DTBZ [vesicular monoamine transporter type-2 (VMAT2)]; [^18^F]FP-CIT [dopamine transporter (DAT)]; H_2_^15^O (brain metabolism); [^11^C]PK11195 (microglial activation), [^18^F]FDG (glucose metabolism); [^18^F]FDDNP (hyperphosphorilated tau); [^11^C]PiB (amyloid deposition); [^11^C]flumazenil (GABA_A_ receptors); [^11^C]diprenorphine (opioid receptors); [^11^C]PMP [acetylcholinesterase (AChE) activity]; and [^11^C]NMPB [muscarinic acetylcholine receptors (mAChRs)]. A total of 77 PET studies were identified and reviewed in this article.

## Results

### Brain metabolism

[^18^F]FDG PET has been employed to assess regional cerebral glucose metabolism as a marker of neuronal activity and neurodegeneration in atypical parkinsonism. In MSA, [^18^F]FDG PET studies have shown reduced brain metabolism in the frontal cortex, striatum, cerebellum, and brainstem [25–32]. Decreased [^18^F]FDG uptake in putamen, brainstem, or cerebellum is part of the diagnostic criteria for possible MSA [33]. Patients with a diagnosis of possible MSA showed greater decreases in glucose metabolism than patients with probable MSA who had severe autonomic dysfunction [34]. These finding suggest that the pattern of glucose metabolism in probable MSA with predominant involvement of the autonomic nervous system, may differ from that of possible MSA, which mainly involves the striatonigral or olivopontocerebellar systems. Glucose hypometabolism mainly occurs in the bilateral putamen in parkinsonian-type and in the bilateral cerebellum in cerebellar-type MSA patients [35] in line with the neuropathologic feature of these two MSA subtypes [36]. MSA patients showed also a different pattern in metabolic changes at different stages of the disease suggesting that cortical hypometabolism begins in the frontal cortex and spreads to the parieto-temporal cortex [37]. Decreases in cerebellar glucose metabolism occur early in the course of the disease and contribute to cerebellar symptoms, whereas motor symptoms precede putaminal hypometabolism [37]. Cerebellar hypometabolism is associated with the severity of ataxia, whereas decreased glucose metabolism in the brainstem correlated with the severity of autonomic dysfunction [31]. Using H_2_^15^O PET, Payoux and colleagues [38] investigated patterns of motor activation before and after an acute levodopa challenge in MSA, PD patients, and a group of healthy controls. Before levodopa challenge, MSA patients showed greater activation of left superior parietal areas and lower bilateral cerebellar activation than healthy controls. In comparison with PD patients, MSA patients had less bilateral cerebellar and greater supplementary motor and left superior parietal activation [38]. Following levodopa challenge, MSA patients exhibited reduced activation in anterior cingulate, whereas patients with PD had greater activation in the right cerebellum. These different patterns of activation may be explained by the presence of cerebellar dysfunction in MSA patients and activation of frontoparietal cortical areas may be seen as compensatory mechanism [38].

In PSP, [^18^F]FDG PET studies showed a significant hypometabolism in bilateral medial frontal cortex involving mainly the anterior and mid cingulate gyri, the supplementary motor area (SMA), the ventro- and dorsolateral premotor areas, the prefrontal areas, the striatum (caudate, in particular), thalamus, and the brainstem [39–46]. Different patterns of glucose metabolism has been observed in PSP subtypes [44]. Richardson’s syndrome (RS) patients showed pronounced thalamic and frontal cortex hypometabolism, whereas PSP-parkinsonism (PSP-P) patients had more significant putaminal hypometabolism Moreover, the putamen/thalamus [^18^F]FDG uptake ratio was able to discriminate between PSP-P and RS patients [47]. Piccini and colleagues [48] studied three affected patients and their asymptomatic relatives from two kindreds with familial PSP. Striatal [^18^F]FDG uptake was reduced in PSP patients in agreement with findings reported for sporadic PSP [48]. Moreover, 33 % of asymptomatic relatives showed cortical and subcortical hypometabolism suggesting the presence of subclinical PSP [48]. [^18^F]FDG PET was used to investigate neural correlates of PSP symptoms [49–52]. A PET study investigated the brain network relevant to postural imbalance and falls in a cohort of 16 PSP patients [50]. Decreased glucose metabolism in the thalamus and increased in the precentral gyrus were associated with severity of postural instability and frequency of falls suggesting that thalamo-cortical circuits are critical for postural imbalance in PSP [50]. Another study from the same group assessed locomotor network in 12 PSP patients [51]. PSP patients underwent a [^18^F]FDG PET at rest and during walking. Hypometabolism was observed at rest in the prefrontal cortex, the subthalamic nucleus, and the pedunculopontine/cuneiform nucleus complex of PSP patients compared to the group of healthy controls [51]. The severity of gait disorder was associated with decreases in glucose metabolism in the prefrontal cortex and subthalamic nucleus. During walking, PSP patients showed decreased [^18^F]FDG uptake in the of the indirect locomotor pathway via the prefrontal cortex–subthalamic nucleus–pedunculopontine/cuneiform nucleus loop, whereas activation of the direct locomotor pathway from primary motor cortex to the spinal cord was observed in PSP patients [51]. These findings suggest that gait dysfunction in PSP is related to impairment of the indirect, modulatory prefrontal–subthalamic–pedunculopontine loop of locomotor control. The increase in the direct locomotor loop may be seen as a compensatory mechanism or contribute to the stereotyped gait pattern in PSP. Oculomotor dysfunction was also associated with reduced regional glucose metabolism in PSP patients [52]. In specific, hypometabolism in the anterior cingulate gyrus was associated with downward gaze palsy. Reduced [^18^F]FDG uptake in the rostral cerebellum was positively correlated with velocity of horizontal saccades and decreased glucose metabolism in the oculomotor vermis was associated with peak velocity of the optokinetic reflex. Smooth pursuit eye movement was correlated with hypometabolism in the inferior parietal and temporal regions and frontal eye field [52]. Reduced glucose metabolism in the frontal cortex was associated with worse Mini Mental Status Examination (MMSE) scores in 16 PSP patients the presence of frontal lobe dysfunction in PSP [49]. Frontal brain regions and in specific cingulate gyrus hypometabolism is also a feature of Frontotemporal Dementia [53–57]. Hypoperfusion of the midcingulate cortex was reported in both PSP and FTD tau patients and correlated with executive dysfunction [58]. In specific, in PSP patients showed hypoperfusion of the posterior part of the midcingulate gyrus, which was associated with worse performance at the Stroop III and Weigl tests [58].

In CBS patients, [^18^F]FDG PET has shown a characteristic asymmetric pattern of impaired glucose metabolism contralateral to the most affected side involving the parietal cortex (usually most prominent), the primary sensorimotor cortex, the medial and lateral premotor areas, the striatum, and the thalamus [35, 44, 46, 56, 59–66]. Cortical metabolic asymmetry was also associated with asymmetries in thalamic [^18^F]FDG uptake suggesting the presence of cortico-thalamic metabolic asymmetry, which is in agreement with focal neuropathological changes reported in CBS [59]. [^18^F]FDG PET was used to investigate the brain networks underlying upper limb praxis processing in a cohort of probable CBS [67]. Anterior cingulate hypometabolism was observed in those CBS patients who performed below the cut-off score at the upper limb apraxia assessment [67]. Moreover, CBS patients who were unable to correct their errors at the same rate as controls showed hypometabolism in superior parietal lobule and SMA suggesting that different neural networks are involved in distinct aspects of the upper limb apraxia in CBS [67].

Cerebral glucose metabolism patterns may be useful to differentiate atypical parkinsonian syndromes. Disease-specific patterns of reduced metabolic activity were found in MSA (bilateral putamen, pons, and cerebellum), PSP (caudate, thalamus, midbrain, and prefrontal cortex), CBS (asymmetrical cortical regions and basal ganglia) [30, 32, 35, 44, 46, 65, 68–72]. The most consistent difference in metabolic activity between atypical parkinsonism and PD is decreased striatal glucose metabolism in atypical parkinsonism patients [30, 32, 35, 42, 47, 56, 68, 72], which is in agreement with post-mortem studies showing post-synaptic striatal neuronal loss in MSA, PSP, and CBS, but not in PD [73].

Specific voxel-based metabolic covariance patterns were able reliably to differentiate PSP, MSA, and CBS patients from healthy controls [66, 69]. An MSA-related pattern was characterized by a significant pattern of metabolic decreases in the putamen and the cerebellum, whereas a PSP-related pattern showed hypometabolism in the brainstem, medial thalamus, caudate nuclei, and medial frontal cortex [69]. A CBS-related pattern involves asymmetrical reductions contralateral to the more affected side in the cerebrum, lateral parietal and frontal regions, and thalamus, with relative bilateral increases in occipital regions [66]. The CBS-related pattern distinguished CBS from MSA, but not PSP patients because of the 24 % overlap existing in CBS and PSP spatial metabolic patters [66]. However, by measuring the degree of hemispheric asymmetry at the network level and comparing with the PSP-related pattern, the authors were able to discriminate between CBS and PSP with 92–94 % specificity [66]. The same group developed an automated image-based classification procedure to differentiate patients with PD, MSA, and PSP [45]. This method showed high sensitivity and specificity in distinguishing these three diseases and may be useful in improving diagnostic accuracy [45]. Visual and SPM analysis of [^18^F]FDG PET showed also higher sensitivity (90–95 %) in differentiating MSA from PD patients than visual MRI analysis [74, 75]. [^18^F]FDG PET studies compared cerebral glucose metabolism of PSP and CBS patients. The most consistently findings were a significantly asymmetrical hypometabolism of the parietal lobe and the primary sensorimotor cortex in CBS patients, while PSP patients usually showed a significantly lower bilateral glucose metabolism of the anterior cingulate gyrus and the upper brainstem [41, 42, 44, 61, 64]. Asymmetric presentation of clinical symptoms in PSP patients may make differential diagnosis between PSP and CBS difficult. A recent PET study investigated differences in glucose metabolism between PSP with asymmetric clinical symptoms, PSP with symmetric presentation, and CBS patients [76]. PSP patients with asymmetrical clinical symptoms showed significant asymmetrical hypometabolism contralateral to the clinically most affected side in ventrolateral thalamus, middle cingulate gyrus, and sensorimotor cortex compared to PSP patients with symmetrical symptoms [76]. In comparison with CBS patients, PSP patients with asymmetrical and symmetrical clinical symptoms showed similar bilateral medial frontal hypometabolism [76]. Asymmetric parietal hypometabolism extending into the premotor cortex contralateral to the clinically most affected side was observed in CBS patients [76]. These findings suggest that disease-specific patterns of [^18^F]FDG uptake may also help with the differential diagnosis between PSP patients with asymmetrical symptoms and CBS.

[^18^F]FDG PET has been used to test the efficacy of treatment in atypical parkinsonism. A small-randomised placebo controlled clinical trial assessed the effects of physostigmine, a cholinesterase inhibitor, on clinical symptoms and brain glucose metabolism in six PSP patients [77]. Intravenous infusion of physostigmine increased by 8 to 32 % regional glucose metabolism and improved ocular movements and attention at the cognitive test [77]. These findings suggest that enhancement of cerebral cholinergic transmission in PSP using physostigmine could increase glucose entry into the brain [77].

### Dopaminergic system

PET studies using [^18^F]dopa, [^18^F]FP-CIT, or [^11^C]DTBZ provided information about the dysfunction of brain monoaminergic projections in patients with atypical parkinsonism. In MSA, [^18^F]dopa uptake was significantly decreased in the caudate, putamen, ventral striatum, and globus pallidus reflecting loss of dopaminergic innervations from the substantia nigra [68, 78–82]. Moreover, significantly decreases in [^18^F]dopa uptake were found in the red nucleus of MSA patients compared to healthy controls suggesting dysfunction of the midbrain tegmentum, locus coeruleus, and median raphe which project to this region [81]. Caudate-putamen index, reflecting differences in the uptakes in the caudate and putamen divided by the caudate uptake, showed greater caudate involvement in MSA in comparison with PD patients suggesting that caudate-purtamen index may be useful in discriminating MSA from PD [80].

Pre-synaptic dopaminergic dysfunction is also associated to clinical symptoms. In specific, reduced [^18^F]dopa uptake in the locus coeruleus correlated with the severity of orthostatic hypotension in MSA patients [81] and striatal [^18^F]dopa decreases was associated with worse locomotor disability [78]. A recent [^18^F]FP-CIT PET study has investigated differences in dopamine transporter (DAT) in three different phenotypes of MSA divided according to different patterns of glucose metabolism: MSA-P_m_ (only striatal hypometabolism), MSA-C_m_ (only cerebellar hypometabolism), and MSA-mixed_m_ (both striatal and cerebellar hypometabolism) [83]. A significant difference in striatal DAT binding was found among the metabolic phenotypes. A marked decreased striatal DAT binding with higher anteroposterior and ventrodorsal gradient was observed in the MSA-P_m_ and MSA-mixed_m_ groups, whereas less decrease was seen in the MSA-C_m_ group [83]. VMAT2 density was significantly decreased in the caudate and putamen in MSA patients [84, 85] and in the cerebellum of MSA-C patients [85]. Moreover, significant correlations were found between decreased striatal binding and the severity of parkinsonian features and between reduced cerebellar binding and severity of cerebellar dysfunction [85]. These findings suggest a differential severity of degeneration of dopaminergic nigrostriatal and non-dopaminergic monoaminergic cerebellar systems in MSA-P and MSA-C subtypes. Decreased striatal [^11^C]DTBZ binding was also associated with the severity of REM sleep behaviour disorder (RBD) in 13 patients with probable MSA [86]. Decreased striatal [^18^F]Fdopa uptake correlated with the severity of extrapyramidal symptoms in MSA [31].

In PSP, [^18^F]dopa uptake was significantly decreased in both caudate and putamen. Differently from PD patients where a rostro-caudal putamen gradient of dopaminergic dysfunction is observed, in PSP, [^18^F]dopa uptake in the caudate and anterior putamen was depressed to the same level as that in the posterior putamen [78]. This pattern of presynaptic degeneration was also observed with [^18^F]FP-CIT PET [87]. Patients with PSP showed more prominent and earlier DAT losses in the anterior caudate and ventral putamen compared to PD patients [87]. Moreover, by applying discriminant function analysis Burn and colleagues [88] found that [^18^F]dopa uptake can reliably discriminate between normal subjects and parkinsonian patients, but differential diagnosis between PD, MSA, and PSP remained difficult. In CBS, [^18^F]Fdopa was markedly reduced with an asymmetry in the caudate and the putamen contralateral to the most affected side [61, 89]. However, single positron emission tomography (SPECT) studies have shown that nigrostriatal degeneration may occur at later stages of the disease [90–95] after cortical hypometabolism took place [93]. In 23 early to mid stages, CBS patients, of whom one had post-mortem confirmed diagnosis of definite CBS, striatal presynaptic DAT uptake was mildly decreased with 39 % of the subjects presenting no dopaminergic deficits [92]. Moreover, postsynaptic dopamine D2 receptor density was preserved [92].

Post-synaptic dopaminergic function has been investigated in atypical parkinsonism with [^11^C]raclopride PET. Studies have shown significant decreases of striatal dopamine D2 receptor density in patients with MSA-P and PSP compared to PD and healthy controls [43, 68, 96–98]. Striatal antero-posterior gradient of [^11^C]raclopride binding was significantly higher in MSA-P than in PD patients [68, 98]. Van Laere and colleagues [98] showed that combined analysis of striatal binding and regional influx of [^11^C]raclopride may aid in differentiating PD from MSA-P patients. PET with [^18^F]DMFP, a fluorine-18 dopamine D2 radioligand, showed reduced striatal uptake in MSA and PSP patients compared to healthy controls and PD patients [99]. Receiver operating characteristic (ROC) curve analysis showed a specificity, sensitivity and accuracy of 100 %, 74 % and 86 %, respectively, of [^18^F]DMFP molecular imaging for the differential diagnosis of atypical parkinsonism and PD [99].

### Microglial activation

Microglia constitute about 10 % of glial cells and are primary mediators of neuroinflammation that respond to pathological changes in the brain producing an excess of various pro-inflammatory cytokines such as TNF-a and IL-1β [100]. These cytokines in turn cause further activation of microglia, resulting in a self-propagating inflammatory cascade, leading to neuronal death and contributing to pathogenesis of atypical parkinsonism [101, 102]. When microglia become activated they overexpress the 18-kd translocator protein (TSPO), which can be detected in vivo with PET and selective radioligands [103, 104]. PET with [^11^C]PK11195 has been employed to study a small cohort of patients with PSP, MSA, and CBS [105–107]. Increased [^11^C]PK11195 binding was observed in the caudate, putamen, pallidum, substantia nigra, midbrain, thalamus, cerebellum, and frontal lobe in four PSP patients [107]. In the two patients who were rescanned after 6–10 months, microglial activation remained stable although patients progressed clinically [107]. Similarly, four patients with CBS showed increased [^11^C]PK11195 binding in caudate, putamen, substantia nigra, and frontoparietal cortex [106].

Increased [^11^C]PK11195 binding was also found in the dorsolateral prefrontal cortex, caudate, putamen, pallidum, thalamus, substantia nigra, and pons of five MSA patients [105]. Moreover, increased pontine microglial activation correlated with MRI measures of tissue damage such as regional apparent diffusion coefficient, although was not associated with putaminal water diffusivity changes in patients with MSA-P and PSP [108]. [^11^C]PK11195 PET has also been used to monitor the efficacy of anti-inflammatory agents therapies in atypical parkinsonism. In a randomized placebo-controlled trial assessing the efficacy of minocycline in patients with MSA, decreased [^11^C]PK11195 binding was observed following treatment, although no clinical benefit of minocycline was found in the trial [109]. Moreover, clinical assessments did not correlate with [^11^C]PK11195 binding. This discrepancy may be due to the small number of participants or to the short duration and/or weakness of the minocycline effects that may be unable to inhibit the pathways downstream of microglial activation leading to the expression of clinical symptoms.

### Other systems

Using PET with [^11^C]diprenorphine, Burn and colleagues [110] investigated the pattern of striatal opioid receptor density in the brain of MSA-P, PSP, and PD patients. They found no significant differences in opioid receptor density in PD patients, whereas decreased [^11^C]diprenorphine binding was observed in the putamen, but not caudate of MSA-P patients and opioid receptor decreases were seen in both caudate and putamen in PSP patients [110]. MSA-C patients showed 85–88 % decreases in opioid receptor density in both caudate and putamen [79]. Foster and colleagues [111] explored the integrity of GABAergic neurons in metabolically affected cortical regions in PSP patients. A reduction of 13 % in [^11^C]flumazenil binding was found in hypometabolic cortical regions, but not in subcortical nuclei, suggesting that loss of cortical neurons containing benzodiazepine receptors may contribute to cerebral cortical hypometabolism in PSP [111]. One PET study using [^11^C]NMPB, a radioligand for mAChRs, has studied cholinergic dysfunction in a cohort of PSP patients with cognitive impairment [112]. Differently to PD, PSP patients showed no significant changes in cortical mAChR levels, and there was no correlation between [^11^C]NMPB binding and cognitive impairment as measured by the MMSE scores [112]. These findings suggest that dysfunction of the cholinergic system may not play a role in cognitive impairment in PSP. PET with [^11^C]PMP showed significant decreases in cortical AChE activity in MSA-P patients at a similar level of those seen in PD patients [113]. In MSA-P and PSP, subcortical cholinergic activity was more decreased than in PD patients with greater involvement of the pontine cholinergic group [113]. Since pontine cholinergic group plays an important role in modulating motor function and in specific gait, this finding may account for greater gait impairment in MSA-P and PSP than PD.

## Conclusion

In recent decades, PET molecular imaging has provided a better understanding of the pathophysiological mechanisms underlying atypical parkinsonism. However, most of the PET imaging studies was performed in patients with probable diagnosis while data in possible MSA or PSP are limited. Moreover, none of the available PET techniques has shown to be a reliable and early biomarker to differentiate between atypical parkinsonism and PD and monitor disease progression and response to treatment. [^18^F]FDG PET has shown higher accuracy than dopaminergic imaging techniques to distinguish among parkinsonian syndromes and in differentiating between atypical parkinsonism. A concomitant loss of pre- and post-synaptic terminals in atypical parkinsonism could explain the limited efficacy of levodopa replacement therapy in these degenerative disorders. [^11^C]PK11195 PET has shown different patterns of microglial activation in atypical parkinsonism, which reflects the known pathology in these disorders. However, [^11^C]PK11195 shows high level of non-specific binding and a poor signal-to-noise ratio [114], which complicates its quantification; moreover, test–retest data in control subjects showed only moderate intra-individual reproducibility [115]. Second generation TSPO ligands for PET imaging may provide a better quantification of microglial activation in atypical parkinsonism. Additionally, astroglial activation plays a key role in the initiation and progression of atypical parkinsonism. PET with specific radioligands targeting astroglial activation may provide new insight in the molecular mechanisms underlying the pathogenesis of these disorders. Recently, a number of fluorinated radioligands have shown in vitro and in vivo ability to label tau aggregates and to possess selectivity for tau over β-amyloid aggregates [116–120]. In vivo imaging of tau aggregates with PET may aid in early diagnosis of CBS and PSP and provide a tool to monitor disease progression. Additionally, tau PET can also serve as a potential biomarker of disease progression and an indicator of treatment efficacy for interventions aimed at preventing tau aggregate formation.
